# Light-emitting diode photobiomodulation therapy for non-specific low back pain in working nurses: A single-center, double-blind, prospective, randomized controlled trial

**DOI:** 10.1097/MD.0000000000021611

**Published:** 2020-08-07

**Authors:** Yen-Po Lin, Ying-Hao Su, Shih-Fang Chin, Yu-Ching Chou, Wei-Tso Chia

**Affiliations:** a National Taiwan University Hospital Hsin-Chu Branch, Hsinchu City; b School of Public Health, National Defense Medical Center, Taipei, Taiwan (R.O.C.).

**Keywords:** low back pain, light-emitting diode, nurse, photobiomodulation

## Abstract

**Background:**

Low back pain (LBP) affects approximately 51% to 57% of hospital nurses and nurses’ aides in Europe. New high-risk groups include home- and long-term-care nurses and physiotherapists. A number of European countries are experiencing a shortage of healthcare workers. Light therapy has been shown to be an effective treatment for various musculoskeletal disorders, including lateral epicondylitis, temporomandibular joint pain, carpal tunnel syndrome, and delayed-onset muscle soreness. A systematic review and meta-analysis demonstrated that low-level laser therapy is an effective method for relieving non-specific chronic low back pain (NSCLBP). However, the efficacy of light-emitting diode (LED) therapy for NSCLBP is disputed. This study aims to evaluate the effect of LED therapy on NSCLBP.

**Methods and analysis:**

We conducted a prospective, double-blind, randomized placebo-controlled trial of 148 patients with NSCLBP. The patients were randomly assigned to 2 groups: intervention group, where patients received LED photobiomodulation therapy 3 times a week for 2 weeks, and the sham group, where patients had sham therapy 3 times a week for 2 weeks. Primary outcome measures included the visual analog scale for pain, lumbar active range of motion assessments, and chair-rising times. Secondary outcome measures included a multidimensional fatigue inventory, fear-avoidance beliefs questionnaire, and the Oswestry disability index. The outcome measures were assessed before therapy and 2weeks, 4 weeks, 8 weeks, 12 weeks, and 6 months after the first interventions were completed.

**Discussion:**

This study is a prospective, single-center, double-blind, randomized, controlled study. This study aims to research the efficacy of a 2-week LED program for NSCLBP working nurse. Our results will be useful for patients, working nurses, nurses’ aides, and other healthcare workers with chronic low back pain.

**Trial registration number:**

NCT04424823

## 1 Introduction

Low back pain (LBP) is the leading cause of disability in both low-income and high-income countries and is associated with large direct and indirect healthcare costs.^[1–3]^ It is the major cause of work absenteeism and musculoskeletal disability.^[4]^ The annual LBP prevalence among hospital nurses and nurses’ aides in Europe is between 51% and 57%, and new high-risk groups include home and long-term care nurses and physiotherapists.^[5]^ A number of European countries are experiencing a shortage of healthcare workers^[6]^; hence, identifying ways to reduce the prevalence of long-term LBP and thus prevent absences associated with LBP among healthcare workers is crucial.

Back pain is generally self-limiting. However, a significant percentage of patients develop chronic back pain.^[7]^ Chronic low back pain (CLBP) is defined as back pain lasting more than 12 weeks. CLBP may be associated with true radicular symptoms in patients with canal or foraminal compromise or pseudoradicular leg pain where the pain does not fall into a particular dermatomal pattern. CLBP is generally assumed to be due to the following:

1. Dysfunction of the motion segment of the spine (discovertebral complex and facets), in which varying degrees of discal degeneration based on MRI are noted^[8]^
2. Non-specific CLBP, which may not show significant degenerative changes in MRI.

Previous studies worldwide have documented a higher LBP prevalence in nursing personnel than in other occupations,^[9–11]^ with an annual prevalence ranging from 45%^[12]^ to 77%.^[13]^ Persistent LBP in nurses causes considerable functional and work disability^[10]^ and is a strong risk factor for long-term sickness absence^[14]^ and dropout from profession at the early stages of their career.^[15]^ Thus, prevention of persistent LBP in nurses is a priority.

Photobiomodulation therapy (PBMT) is a non-pharmacological intervention often used in the treatment of musculoskeletal disorders, such as LBP.^[16–19]^ Photobiomodulation therapy consists of applying a non-ionized form of light, which includes light amplification by stimulated emission of radiation or commonly known as laser, light-emitting diodes (LED), and other lights with a broader spectrum, ranging from visible to infrared. ^[20]^ Light energy exerts biochemical, bioelectrical, bioenergetic, and biostimulatory effects.^[21]^ Mechanisms by which light therapies have been shown to relieve pain include increases in microcirculation and nitric oxide synthesis, enhanced release of endorphins, modulation of nerve transmissions, and modulation of key mediators of inflammation, such as inhibitory cyclooxygenase and prostaglandin E2.^[22]^ Because it promotes tissue healing and produces anti-inflammatory and analgesic effects, light therapy is commonly used to treat musculoskeletal conditions.^[23–26]^ Moreover, light therapy has been shown to be an effective treatment for various musculoskeletal disorders, including lateral epicondylitis,^[27]^ temporomandibular joint pain,^[24]^ carpal tunnel syndrome,^[28]^ and delayed onset muscle soreness.^[23]^ A previous systematic review and meta-analysis including 221 studies and 7 randomized controlled trials demonstrated that low-level laser therapy is an effective method for relieving non-specific chronic low back pain (NSCLBP).^[29]^ Foley et al showed that 830-nm LED phototherapy significantly reduced adverse advents in return to play of university athletes with a wide range of injuries.^[30]^ Currently, few studies have investigated the use of bright light or LED therapy in Carvalho et al showed that LED therapy in lateral decubitus position and with flexion exercises of the lower limbs for the treatment of L4-L5 and L5S1 lumbar disk herniation with radiculopathy showed better therapeutic performance for radicular pain, gait claudication, and functional disability.^[31]^ In addition, Hsieh and Lee showed that treatment with hot-pack therapy and 890-nm light therapy is associated with reductions in the severity of disability and fear avoidance beliefs in patients with CLBP.^[32]^ The use of 890-nm light therapy was approved by the United States Food and Drug Administration for the treatment of minor muscle and joint pain in 2002.^[33]^ Moreover, previous studies have shown that 890-nm light therapy reduces pain^[34]^ without detrimental systemic cardiovascular effects.^[35]^

Thus, this double-blind randomized controlled trial aimed to evaluate the effect of LED PMBT on pain, fatigue, fear avoidance beliefs, and function in individuals, particularly nurses, with NSCLBP. We hypothesized that LED PMBT 3 times a week for 2 weeks would be effective in reducing pain and fear of pain among female nurses with NSCLBP.

## 2 Methods

### 2.1 Study design

**Figure 1:**
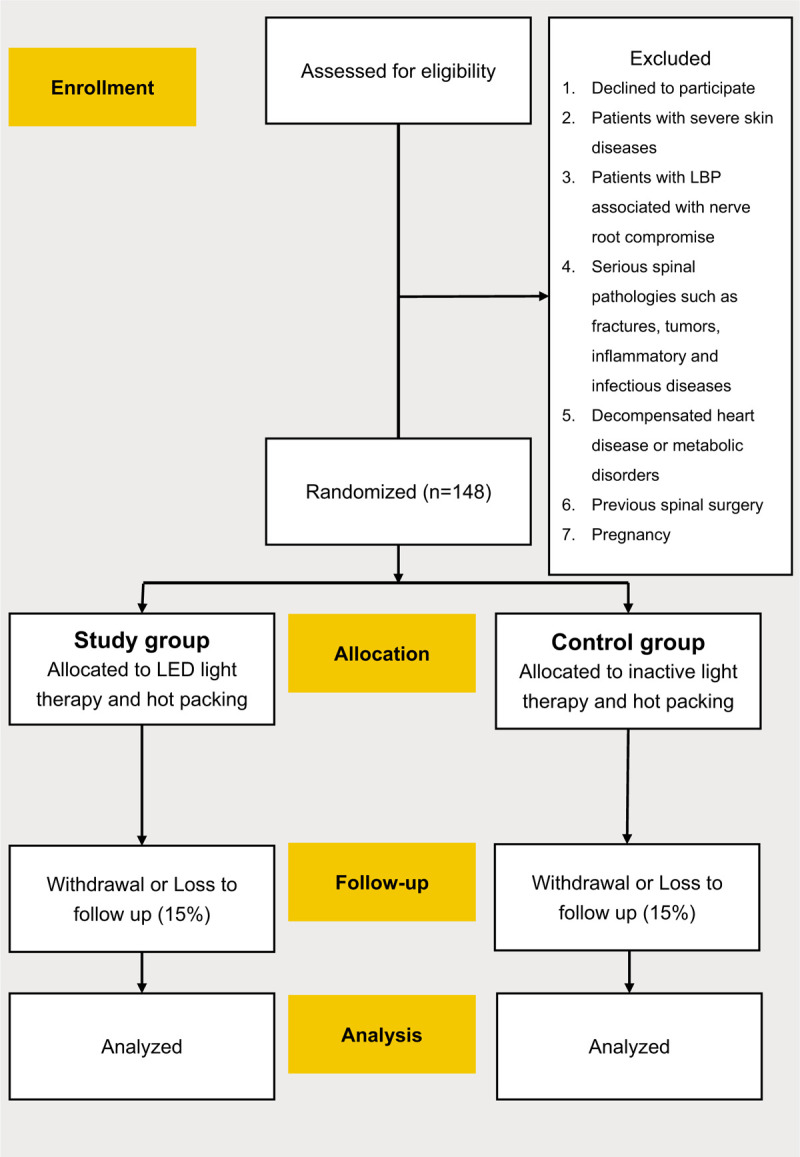
The study protocol is in accordance with the 2013 SPIRIT statement.

The study protocol is in accordance with the 2013 SPIRIT (Standard Protocol Items: Recommendations for Interventional Trials) Statement (see Additional file 1 for the populated SPIRIT Checklist). Figure 1 shows the enrollment of the subjects, interventions, and assessments according to SPIRIT. This randomized, double-blind (patients and outcome assessors), prospective, controlled clinical trial was performed at the orthopedics department of National Taiwan University Hospital Hsin-Chu Branch. This study received approval from the Human Research Ethics Committee of National Taiwan University Hospital Hsin-Chu Branch (process number 108-088-F). All study participants received information regarding the objectives and procedures and signed a written informed consent prior to participation, as stipulated in Resolution 91C-27-0014 of the National Taiwan University Hospital Hsin-Chu Branch.

Participants were randomly allocated to 2 groups: intervention group (participants received LED light therapy) or sham group (participants received placebo interventions, i.e., inactive light therapy). The LED and sham interventions were performed using the same device and the same irradiated sites 3 times a week (with a minimum interval of 24 hours) for 2 consecutive weeks. To ensure blinding of patients, the device was set to produce the same sounds and display the same information during both therapies. Furthermore, because the device produces a non-significant amount of heat, the participants could not determine whether active or shame light therapy was administered. The group assignments were not revealed to the participants.

### 2.2 Participants

Male and female nurses aged 18 to 65 years with a clinical diagnosis of NSCLBP based on history and clinical examination were included in this study. No restrictions were imposed with regard to race.

The inclusion criteria included the following: male or female registered nurses aged 18 to 65 years with non-specific chronic LBP, which is defined as pain or discomfort between the costal margins and inferior gluteal folds with or without referred pain to the lower limbs, and persistent LBP for at least 3 months.^[36]^ The exclusion criteria were as follows: severe skin diseases (e.g., skin cancer, erysipelas, severe eczema, severe dermatitis, severe psoriasis, and severe hives lupus); LBP associated with nerve root compromise (measured by clinical examination of dermatomes, myotomes, and reflexes)^[37,38]^; serious spinal pathologies, such as fractures, tumors, and inflammatory and infectious diseases; decompensated heart disease or metabolic disorders; previous spinal surgery; and pregnancy. Relevant concomitant care and interventions which is same as previous treatment that are permitted.

Moreover, patients with CLBP were allowed or asked to withdraw from the study if patient makes such a request; the patient develops a serious disease, such as stroke or heart disease; and the patient experiences an adverse effect related to the whole body vibration exercise.

### 2.3 Sample calculation

Sample calculation was performed to detect a 1-point difference for the outcome pain intensity (as measured by the Pain Numerical Rating Scale),^[39]^ with an estimated standard deviation of 1.84 points. A statistical power of 80% was considered for the 2 outcomes, with an α level of 5% and a possible sample loss of up to 15%. Therefore, a total of 148 patients were required for this study.

Step 1 – Recruitment of study participants. Patients received information regarding the objectives and procedures, and those who agreed to participate were asked to sign a written informed consent prior to participation.

Step 2 – The participants were blinded to the study group allocation. The therapists were aware of the participants’ group assignment.

### 2.4 Randomization and masking

Prior to the initiation of treatment, the patients were randomized to their respective intervention groups. The randomization was generated by a computer program (MS Excel Office 2010, Microsoft) and performed by a researcher not involved in the recruitment or evaluation of the patients.

### 2.5 Interventions

Patients were submitted to photobiomodulation therapy with wavelengths of both 630-nm and 850-nm for RED and near-infrared LEDs, with power density set to 8 mW/cm^2^ and 14 mW/cm^2^, respectively. The LED device (name of device, Applied BioPhotonics) was designed in Silicon Valley, United States, and was approved by the United States Food and Drug Administration for the treatment of minor muscle and joint pain.^[40]^ Parameters of the LED phototherapies used in this study are described in Table 1. LED therapy was applied by placing the device on the skin at a 90° angle. Both groups (intervention and sham) underwent 6 LED therapy sessions (i.e., 3 times a week for 2 weeks), and during the therapy, only the researcher in charge of programming the LED device was aware of the treatment employed; the programmer did not participate in the execution of the treatments, evaluations, or data analysis.

**Table 1:**
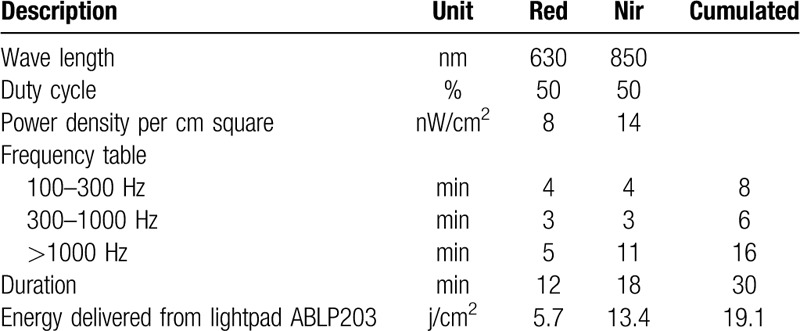
The parameters of the light-emitting diode phototherapies used in this study.

### 2.6 Participants

Age, sex, education level, marital status, work status, smoking, and drinking habits, and comorbidities were recorded for all participants, and their body mass index was calculated.

### 2.7 Outcome measures

The outcome measures were assessed before, 2weeks, 4 weeks, 8 weeks, 12 weeks, and 6 months after the first interventions were completed. The investigator who performed the therapy was blinded to the allocation of each participant.

Primary outcome measures included the following:

1. Lumbar active range of motion assessments, which included forward flexion, extension, and right and left rotations and were measured in degrees using a back range of motion instrument.^[41]^
2. A 100-mm visual analog scale (VAS), which was used for LBP assessment. The anchor terms of the VAS were 0 (no pain) and 10 (maximum pain imaginable). Higher VAS scores indicate greater pain intensity.
3. Chair-rising time, wherein the time required for participants to rise 5 times from a seated position in a standard chair to a standing position as quickly as possible, without using their arms for support,^[42]^ was measured. A longer chair-rising time represents greater physical function limitations.

Secondary outcome measures were assessed with:

1. A multidimensional fatigue inventory (MFI), which was used to assess fatigue.^[43]^ The MFI contains 20 visual 5-point Likert statements that cover different aspects of fatigue, including general fatigue, physical fatigue, reduced activity, reduced motivation, and mental fatigue. A higher MFI score indicates greater fatigue.
2. A fear-avoidance beliefs questionnaire (FABQ), which was used to measure fear-avoidance beliefs regarding physical activity and work.^[44]^ The FABQ is a 16-item questionnaire with 2 subscales: the FABQ physical activity subscale, which contains 4 items that assess fears, avoidance attitudes, and beliefs regarding general physical activity, and the FABQ work subscale, which contains 7 items that assess fears, avoidance attitudes, and beliefs regarding occupational activity. Higher scores in the physical activity (range, 0–24) and work (range, 0–42) subscale evaluations indicate greater fears, avoidance attitudes, and beliefs.
3. An Oswestry disability questionnaire (ODQ), which was used to evaluate the extent of the effect of LBP on the participants’ ability to manage activities of daily living.^[45]^ The ODQ contains 10 questions (total scores ranging from 0–100). Considering that certain cultural differences may be inherent in a questionnaire that was originally developed for a Western population, severity in the ODQ in our study was classified into 5 categories based on the total scores, according to a previous study on CLBP conducted in a Taiwanese population,^[46]^ as follows: minimal disability (0–11), moderate disability (12–22), severe disability (23–32), crippled (33–43), and bed bound (≥44).^[46]^

### 2.8 Statistical methods

Results of our evaluations are expressed as mean ± standard deviation. Chi-squared tests or *t* tests were used to compare the differences in the data between the treatment and placebo groups according to demographic and baseline variables. Paired t tests were used to compare the effects between the intervention and sham groups based on the primary and secondary outcome measures. The level of statistical significance was set at *P* < .05.

## 3 Discussion

Recently, a trend toward the use of LED light-therapy devices for pain relief among medical practitioners has emerged because of the lower costs in the irradiation of large-surface areas compared with treatments using laser-based light-therapy devices.^[22,41,47]^ Theoretically, light therapy should be an effective treatment for NSCLBP. However, the evidence for LED PMBT is inadequate, and the effectiveness of LED in LBP remains to be established. We evaluated the short-term effect of LED therapy using a LED-based device on NSCLBP in this prospective, double-blind randomized placebo-controlled study. This study revealed that LED therapy could be an effective alternative treatment for NSCLBP by reducing pain and fatigue and improving fear-avoidance beliefs, function, and quality of life.

### 3.1 Strengths and limitations

LED therapy is easy to perform, non-invasive, and has no obvious side effects. The limitations of this study include difficult recruit busy working nurse for complete treatment and follow up.

## Acknowledgments

The authors would like to thank the assistant of the department of nursing of National Taiwan University Hospital Hsin-Chu Branch.

## Author contributions

**Conceptualization:** Wei-Tso Chia.

**Data curation:** Ying-Hao Su, Shih-Fang Chin.

**Formal analysis:** Wei-Tso Chia, Yu-Ching Chou.

**Funding acquisition:** Wei-Tso Chia.

**Investigation:** Yen-Po Lin, Shih-Fang Chin.

**Methodology:** Ying-Hao Su, Wei-Tso Chia.

**Resources:** Wei-Tso Chia.

**Supervision:** Wei-Tso Chia.

**Writing – original draft:** Yen-Po Lin.

**Writing – review & editing:** Ying-Hao Su, Wei-Tso Chia.
